# Cold acclimation triggers major transcriptional changes in *Drosophila suzukii*

**DOI:** 10.1186/s12864-019-5745-7

**Published:** 2019-05-22

**Authors:** Thomas Enriquez, Hervé Colinet

**Affiliations:** Université de Rennes1, CNRS, ECOBIO – UMR 6553, 263 avenue du Général Leclerc, 35042 Rennes, France

**Keywords:** Thermal plasticity, Cold tolerance, Transcriptomics, Genes expression, Spotted wing drosophila

## Abstract

**Background:**

Insects have the capacity to adjust their physiological mechanisms during their lifetime to promote cold tolerance and cope with sublethal thermal conditions, a phenomenon referred to as thermal acclimation. The spotted wing drosophila, *Drosophila suzukii*, is an invasive fruit pest that, like many other species, enhances its thermotolerance in response to thermal acclimation. However, little is known about the underlying mechanisms of this plastic response. Here, we promoted flies’ cold tolerance by gradually increasing acclimation duration (i.e. pre-exposure from 2 h to 9 days at 10 °C), and then compared transcriptomic responses of cold hardy versus cold susceptible phenotypes using RNA sequencing.

**Results:**

Cold tolerance of *D. suzukii* increased with acclimation duration; the longer the acclimation, the higher the cold tolerance. Cold-tolerant flies that were acclimated for 9 days were selected for transcriptomic analyses. RNA sequencing revealed a total of 2908 differentially expressed genes: 1583 were up- and 1325 were downregulated in cold acclimated flies. Functional annotation revealed many enriched GO-terms among which ionic transport across membranes and signaling were highly represented in acclimated flies. Neuronal activity and carbohydrate metabolism were also enriched GO-terms in acclimated flies. Results also revealed many GO-terms related to oogenesis which were underrepresented in acclimated flies.

**Conclusions:**

Involvement of a large cluster of genes related to ion transport in cold acclimated flies suggests adjustments in the capacity to maintain ion and water homeostasis. These processes are key mechanisms underlying cold tolerance in insects. Down regulation of genes related to oogenesis in cold acclimated females likely reflects that females were conditioned at 10 °C, a temperature that prevents oogenesis. Overall, these results help to understand the molecular underpinnings of cold tolerance acquisition in *D. suzukii*. These data are of importance considering that the invasive success of *D. suzukii* in diverse climatic regions relates to its high thermal plasticity.

**Electronic supplementary material:**

The online version of this article (10.1186/s12864-019-5745-7) contains supplementary material, which is available to authorized users.

## Background

The spotted wing drosophila, *Drosophila suzukii*, is a fruit fly originating from South-East Asia, invasive in Europe as well as North and South America [1] where it is continuously expending its repartition area [2, 3]. Contrary to its relative species *Drosophila melanogaster,* which lays eggs exclusively on rotten fruits, *D. suzukii* females possess a serrated ovipositor that allows to break through fruit skin and lay eggs in fresh mature fruits [4]. After hatching, the larvae consume the fruits, causing damages that turn them uncommerciable [5, 6]. Furthermore, wounds caused by female’s ovipositor offer a way of entrance for pathogens, causing secondary infections [6]. This fly is highly polyphagous, targeting a broad range of cultivated fruit crops [1, 5, 6], as well as wild hosts [7, 8]. Consequently, this pest has an important economic impact, especially for soft fruit production [1, 5, 6]. In order to facilitate the control of *D. suzukii*, knowledge about its biology is highly required, especially about its thermal physiology [1, 9, 10]. Thermal tolerance and especially the capacity of alien species to modulate their thermal tolerance thanks to phenotypic plasticity, is believed to be a key factor of their invasive success [11, 12]. Therefore, increasing knowledge about thermal biology of *D. suzukii* is essential to predict evolution of its invasion front or its population dynamics in invaded areas and facilitate its control.

Like most insect species [13], *D. suzukii* is chill susceptible which means that it rapidly suffers chilling injuries at temperatures well above its freezing point [14–17]. In insects, chilling injuries result from complex physiological alterations such as loss of ion and water homeostasis which participate to the disruption of neuromuscular functions, leading to chill coma. Physiological injuries also compromise cell integrity, resulting in tissue damage, and in most extreme cases death [13, 18, 19]. To cope with these deleterious effects, insects can adjust their physiological state in anticipation of cold stress. Cold acclimation (triggered by pre-exposure to mild low temperature) is a typical example of phenotypical plasticity. Cold acclimation induces deep and complex physiological remodeling such as changes in composition of membranes [20], mobilization of cryoprotective metabolites [21–24], maintenance of metabolic homeostasis [25–28], altered stress genes expression [29, 30], and enhanced ability to maintain ion and water balance [29, 31, 32]. These changes prevent the development of chilling injuries, resulting in increased cold tolerance [13].

*Drosophila suzukii* displays a high plasticity of cold tolerance and responds to all forms of acclimation such as rapid cold hardening [33, 34], adult acclimation [25, 35, 36] and developmental acclimation [25, 34, 37, 38]. This fly is capable of surviving a three days exposure at − 7.5 °C after dynamic acclimation (i.e. gradual cooling) [39]. Despite the economic importance of this species, the number of studies that focused on the underlying mechanisms of this plasticity remains scarce. Shearer et al., [37] explored transcriptional adjustments associated with the winter-phenotype generated by a combination of developmental and adult acclimation. This thermal treatment results in a cold tolerant winter phenotype showing a reproductive dormancy. In a previous work [25], we showed that flies subjected to both developmental and adult acclimation were characterized by the accumulation of cryoprotectants and were able to maintain metabolic homeostasis after cold stress, suggesting a deep biochemical remodeling linked to acclimation. So far, there is a limited understanding of the molecular mechanisms that underlie cold tolerance plasticity of *D. suzukii.*

In the present study, we subjected mature adults of *D. suzukii* to increasing acclimation periods (pretreatment from 2 h to 9 days at 10 °C) in order to investigate the cold pre-exposure period needed to reach high cold tolerance. Next, we identified the molecular correlates underlying cold tolerance acquisition in *D. suzukii* using the hypothesis-generating and explorative power of RNA sequencing (RNAseq). We expected to find regulations of candidate gene sets involved in the canonical cold-acclimation mechanisms, such as membrane remodeling, cryoprotectant (sugar) metabolism, ionic/water balance or stress proteins.

## Results

### Cold tolerance increased with acclimation duration

**Fig. 1:**
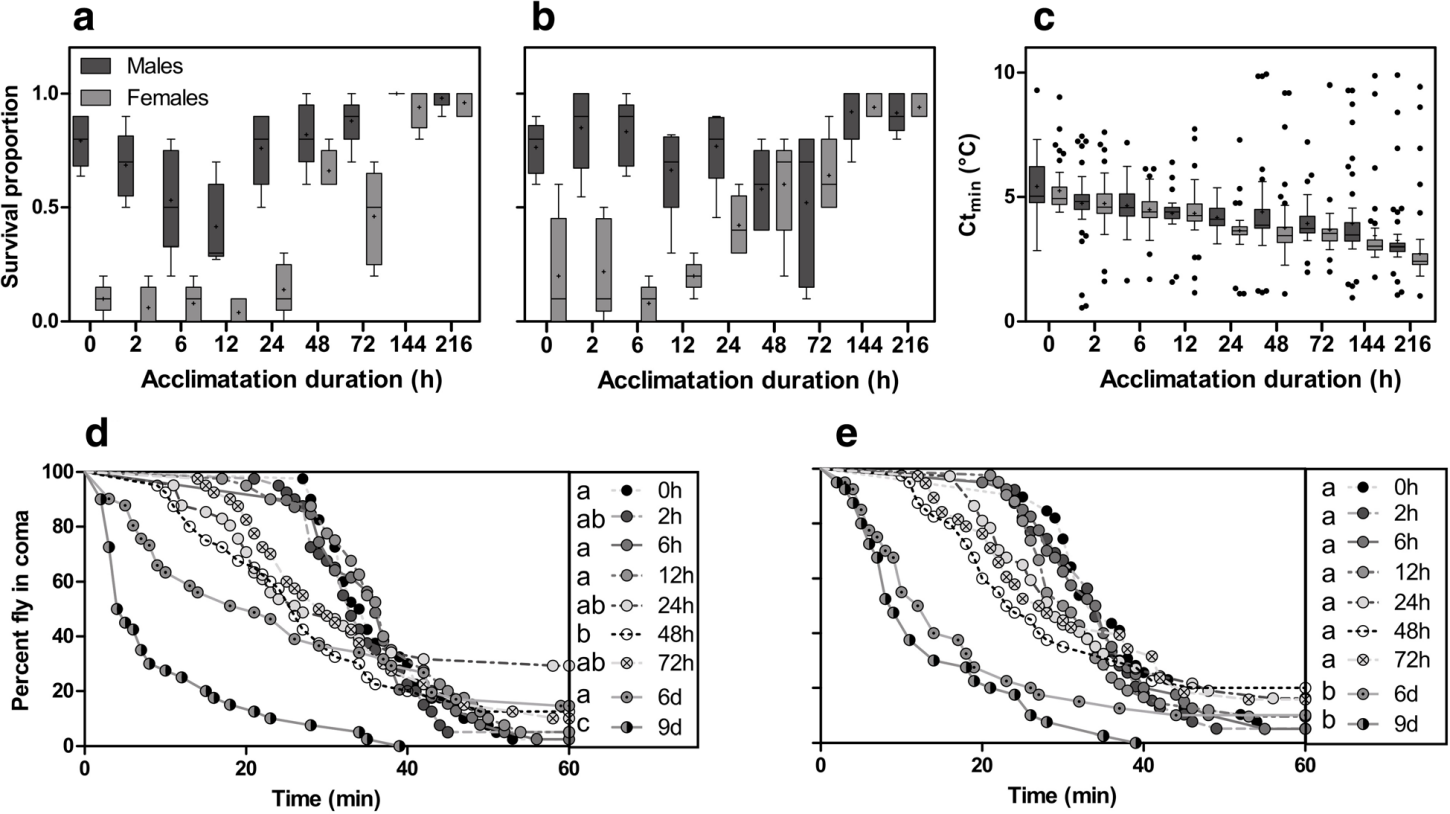
Cold tolerance assays on *Drosophila suzukii* males and females. Flies were either non-acclimated or cold acclimated at 10 °C for incremental durations (from 2 to 216 h). Males (dark grey) and females (light grey) survival after (**a**) an acute cold stress at − 5 °C for 60 min or after (**b**) a chronic cold stress at 0 °C for 24 h according to cold acclimation duration. (**c**) Boxplots describing mean critical thermal minimum (Ct_min_) according to cold acclimation duration for males (dark grey) and females (light grey). Chill coma recovery time (CCRT) following exposure to 0 °C for 12 h according to acclimation duration (indicated in right panel) for males (**d**) and females (**e**). Groups with the same letter in the right panel are not significantly different (Gehan-Breslow-Wilcoxon test to compare survival curves, *p.value* < 0.0013)

Five-day old flies were either cold-acclimated at 10 °C for various durations (2 h, 6 h, 12 h, 24 h, 48 h, 72 h, 6 days, i.e. 144 h or 9 days, i.e. 216 h) or non-acclimated (0 h); this generated nine treatments of acclimation. Survival to acute (− 5 °C for 1 h) and to chronic cold stress (0 °C for 24 h) as a function of acclimation is displayed in Fig. 1a and b, respectively. Regardless of sex, cold survival reached a maximum after 144 h (6 days) of acclimation (Fig. 1a and b). Globally, both acute or chronic cold stress survival increased with acclimation duration, reaching 98% for males and 96% for females after 216 h (9 days) of acclimation (acute: *χ*^2^ = 278.52, *p.value* < 0.001; chronic: *χ*^2^ = 135.10, *p.value* < 0.001; Fig. 1a and b; Additional file 1: Figure S1). However, variations in survival rates were observed, for instance in males, survival was lower after 12 h of acclimation than after 6 h of acclimation (Fig. 1a and b). Overall, males showed a higher survival to acute and chronic cold stress than females (acute: *χ*^2^ = 253.34, *p.value* < 0.001; chronic: *χ*^2^ = 91.509, *p.value* < 0.001; Fig. 1a and b; Additional file 1: Figure S1). Females clearly showed improved cold survival with acclimation duration, whereas benefits in males were much less manifested due to their already-high basal tolerance (Additional file 1: Figure S1). These distinct patterns resulted in significant acclimation duration x sex interaction (acute: *χ*^2^ = 10.75, *p.value* < 0.01; chronic: *χ*^2^ = 48.70, *p.value* < 0.001; Fig. 1a and b).

Mean critical thermal minimum (Ct_min_) of males and females are displayed in Fig. 1c. With no acclimation treatment (i.e. 0 h of acclimation), mean Ct_min_ values were 5.4 ± 0.15 and 5.2 ± 0.11 °C for males and females, respectively. Values gradually decreased with acclimation duration to reach 3.2 ± 0.18 and 2.7 ± 0.12 °C after 216 h acclimation, in males and females respectively (*χ*^2^ = 220.95, *p.value* < 0.001). Ct_min_ values decreased similarly with acclimation in males and females (acclimation duration x sex interaction: *χ*^2^ = 2.09, *p.value* = 0.14).

Chill coma recovery time (CCRT) curves are shown in Fig. 1d and e, for males and females respectively. All statistics comparing the CCRT curves of the different treatments (Gehan-Breslow-Wilcoxon tests) are available in Table 1. All flies from all treatments were in chill coma after 12 h at 0 °C, but the recovery dynamics varied greatly with acclimation treatments. Globally, recovery time decreased with acclimation duration for both males and females, with non-acclimated flies showing the slowest recovery dynamics, and 216 h (9 days) acclimated flies the fastest.

**Table 1:** Comparisons of CCRT (chill coma recovery time) curves between the different acclimation durations, for males and females

Sex / acc. Duration		2 h		6 h		12 h		24 h		48 h		72 h		144 h		216 h	
		*p.value*	*χ* ^2^	*p.value*	*χ* ^2^	*p.value*	*χ* ^2^	*p.value*	*χ* ^2^	*p.value*	*χ* ^2^	*p.value*	*χ* ^2^	*p.value*	*χ* ^2^	*p.value*	*χ* ^2^
Males	0 h	0.27	1.20	0.88	0.02	0.37	0.77	0.11	2.48	0.0004*	12.4	0.03	4.33	0.0027	8.99	< 2.7E-05 ***	60.78
	2 h	/	/	0.44	0.57	0.10	2.70	0.16	1.90	0.004	8.29	0.13	2.20	0.0076	7.12	< 2.7E-05 ***	58.07
	6 h	/	/	/	/	0.55	0.34	0.13	2.19	0.0012*	10.5	0.05	3.82	0.0048	7.96	< 2.7E-05 ***	59.91
	12 h	/	/	/	/	/	/	0.12	2.32	0.0005*	12.2	0.02	4.77	0.0022	9.39	< 2.7E-05 ***	61.14
	24 h	/	/	/	/	/	/	/	/	0.27	1.17	0.85	0.034	0.020	5.37	< 2.7E-05 ***	46.00
	48 h	/	/	/	/	/	/	/	/	/	/	0.15	1.98	0.15	2.01	< 2.7E-05 ***	39.84
	72 h	/	/	/	/	/	/	/	/	/	/	/	/	0.03	4.62	< 2.7E-05 ***	49.98
	144 h	/	/	/	/	/	/	/	/	/	/	/	/	/	/	< 2.7E-05 ***	19.69
Females	0 h	0.47	0.51	0.40	0.70	0.03	4.29	0.04	3.97	0.003	8.38	0.04	3.88	< 2.7E-05 ***	33.49	< 2.7E-05 ***	55.57
	2 h	/	/	0.95	0.003	0.20	1.59	0.14	2.08	0.01	5.85	0.18	1.73	< 2.7E-05 ***	30.85	< 2.7E-05 ***	51.31
	6 h	/	/	/	/	0.24	1.32	0.17	1.83	0.01	5.87	0.17	1.82	< 2.7E-05 ***	31.58	< 2.7E-05 ***	52.56
	12 h	/	/	/	/	/	/	0.43	0.60	0.02	4.90	0.28	1.14	< 2.7E-05 ***	31.00	< 2.7E-05 ***	50.1
	24 h	/	/	/	/	/	/	/	/	0.13	2.24	0.58	0.29	< 2.7E-05 ***	24.63	< 2.7E-05 ***	38.99
	48 h	/	/	/	/	/	/	/	/	/	/	0.13	0.82	< 2.7E-05 ***	15.83	< 2.7E-05 ***	29.02
	72 h	/	/	/	/	/	/	/	/	/	/	/	/	< 2.7E-05 ***	19.78	< 2.7E-05 ***	34.51
	144 h	/	/	/	/	/	/	/	/	/	/	/	/	/	/	0.18	1.75

CCRT curves are available on Fig. 1d and e. *p. values* has been adjusted using Bonferroni correction: * < 0.0013; ** < 0.0002; *** < 2.7E-05 (Gehan-Breslow-Wilcoxon tests)

Additional cold tolerance assays were performed to account for any physiological age distortion between control and acclimated flies. Indeed, the transcriptomic analysis were based on a comparison between control flies versus flies acclimated for 9 days at 10 °C. Even though aging is likely very limited at 10 °C, we reasoned that physiological age of flies might be slightly different between control flies (5d-old mature flies) and acclimated flies (5d-old mature flies + 9 days at 10 °C). Using the developmental zero at which metabolic activity is supposed to stop (7.2 °C in *D. suzukii*, [40]), we estimated that degree days (DD) accumulated during the acclimation period (i.e., 25 DD for 9 days at 10 °C) would be less than two days at 25 °C (i.e., 35 DD). Therefore, we compared cold tolerance (CCRT) of two control fly sets, one of 5d-old and the other of 7d-old, with the cold tolerance of acclimated flies (5d-old + 9 days at 10 °C). We confirmed that acclimated flies were much more cold tolerant than controls regardless of age (Additional file 1: Figure S2).

### RNA sequencing results and qPCRs validation of gene expressions

**Fig. 2:**
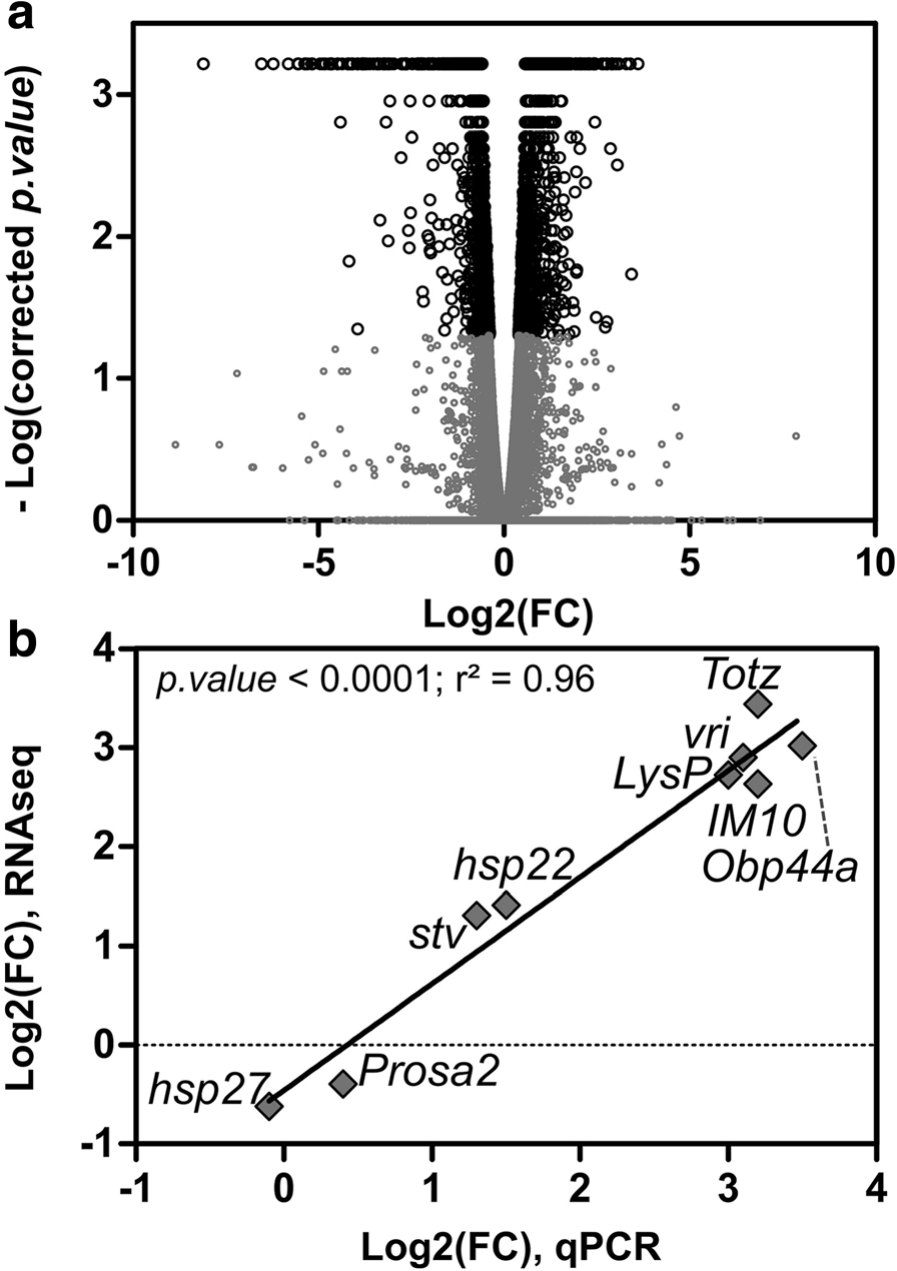
(**a**) Volcano plot of genes expression from RNAseq. Data are plotted as a function of log2 fold change (FC) on X-axis and -log of corrected *p.value* on Y-axis. Black circles correspond to genes with a corrected *p*.*value* < 0.05, and grey circles to genes with a corrected *p.value* > 0.05. Fold change (FC) was calculated using the ratio of expression acclimated/control, so positive values correspond to upregulation in acclimated flies. (**b**) Expression values of nine selected genes based on RNAseq (Y-axis) and qPCRs (X-axis). Expressions resulting from both techniques were highly similar resulting in a significant linear relation with a slope not different form 1 (see results)

From six libraries, comprising three true replicates (i.e. independent pools of 10 flies) of control females (COF1–3) and three of cold acclimated females (CAF1–3), we obtained a total of approximatively 198 million paired end reads, with an average Q30 of 95.13%. After trimming, we obtained approximatively 180 million paired end reads. The mapping resulted in a mean of 71.11% mapped reads (Table 2). A total of 13,486 genes were annotated and used for differential expression analysis. This analysis highlighted 1583 up- and 1325 downregulated genes in cold acclimated flies, among them 378 were expressed exclusively in cold acclimated flies, and 331 only in control flies (Fig. 2a, Additional file 2: Tables S1 and S2). Expression patterns were validated with qPCRs on a selection of nine up- and downregulated genes (Fig. 2b) and were highly similar to expression levels resulting from the differential expression analysis of RNAseq (Spearman correlation: *p.value* < 0.01; linear regression: *p.value* < 0.001; r^2^ = 0.96). The slope of this relation (0.89 ± 0.07) was not different from 1 (*F* = 2.37; *p.value* = 0.14).

**Table 2:** Summary of RNA sequencing metrics

Sample	Yield (Mbp)	N Reads	%Q30	Mean Q	N Reads after trimming	% Mapping
CAF1	8.297	33,187,088	94.98	35.76	30,158,494	71.4
CAF2	7.166	28,665,463	94.84	35.7	26,025,539	70.9
CAF3	6.782	27,126,385	94.96	35.75	24,736,334	71.5
COF1	9.01	36,040,513	95.38	35.8	32,867,578	69.7
COF2	11.272	45,089,307	95.29	35.78	41,064,935	70.2
COF3	6.922	27,686,685	95.38	35.81	25,285,497	73

*Yield (Mbp)* number of bases in mega bases, *Q* quality score, *%Q30* percentage of bases with a quality score of at least 30. *CAF* cold acclimated females, *COF* Control Females

### Gene ontology (GO) terms enrichment on differentially expressed genes revealed implications of several physiological functions in cold acclimated flies

**Fig. 3:**
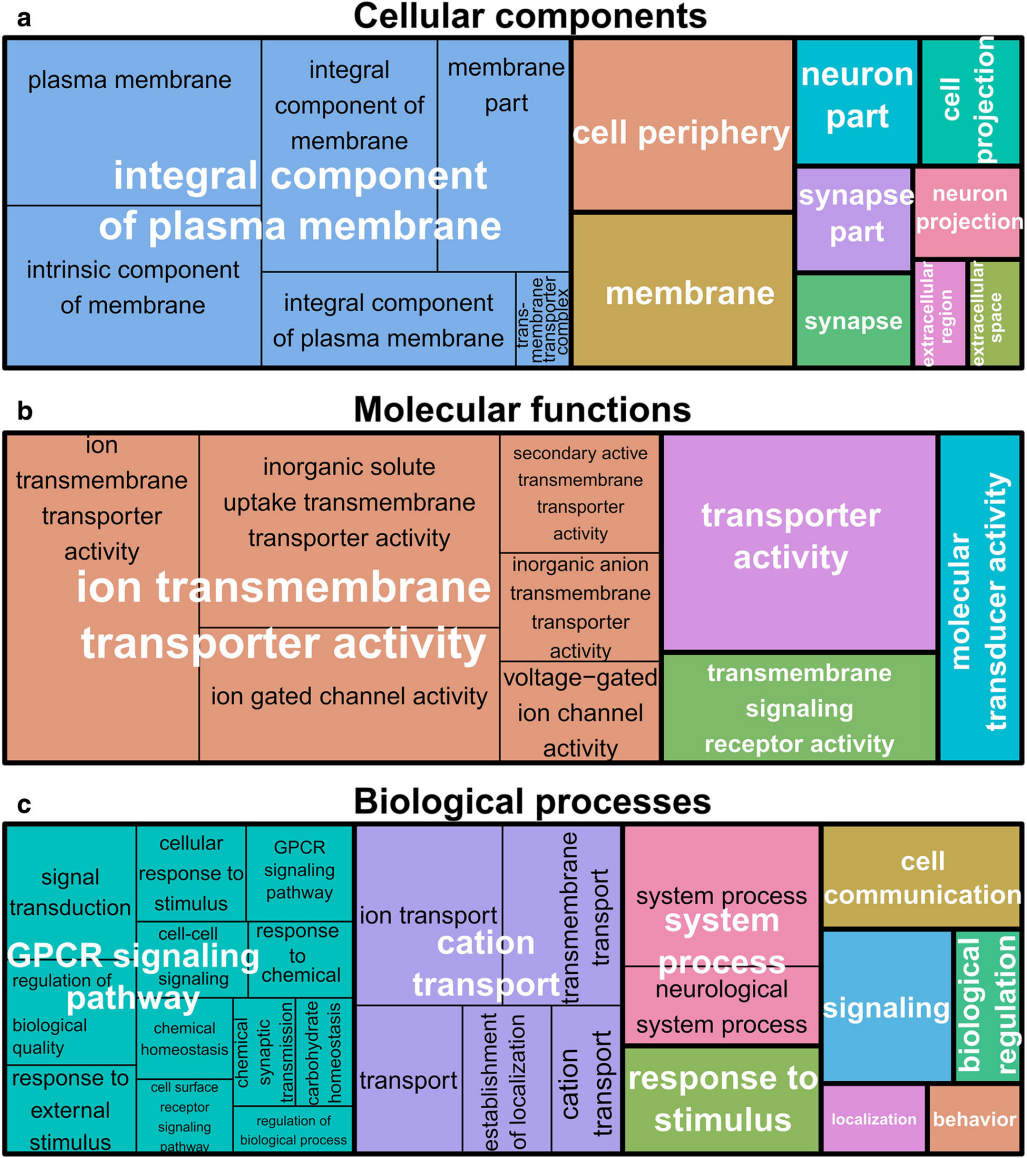
Treemap representation from REVIGO of overrepresented GO-terms from upregulated genes in acclimated flies for: (**a**) cellular components, (**b**) molecular functions and (**c**) biological processes. In each treemap, each rectangle represents a significant GO-term. The sizes of rectangles are adjusted to reflect the relative corrected *p-value* (i.e. the larger the rectangle, the more significantly the GO-term was). Within the treemaps, GO-terms sharing the same color belong to the same GO superclusters whose names are labelled in white

**Fig. 4:**
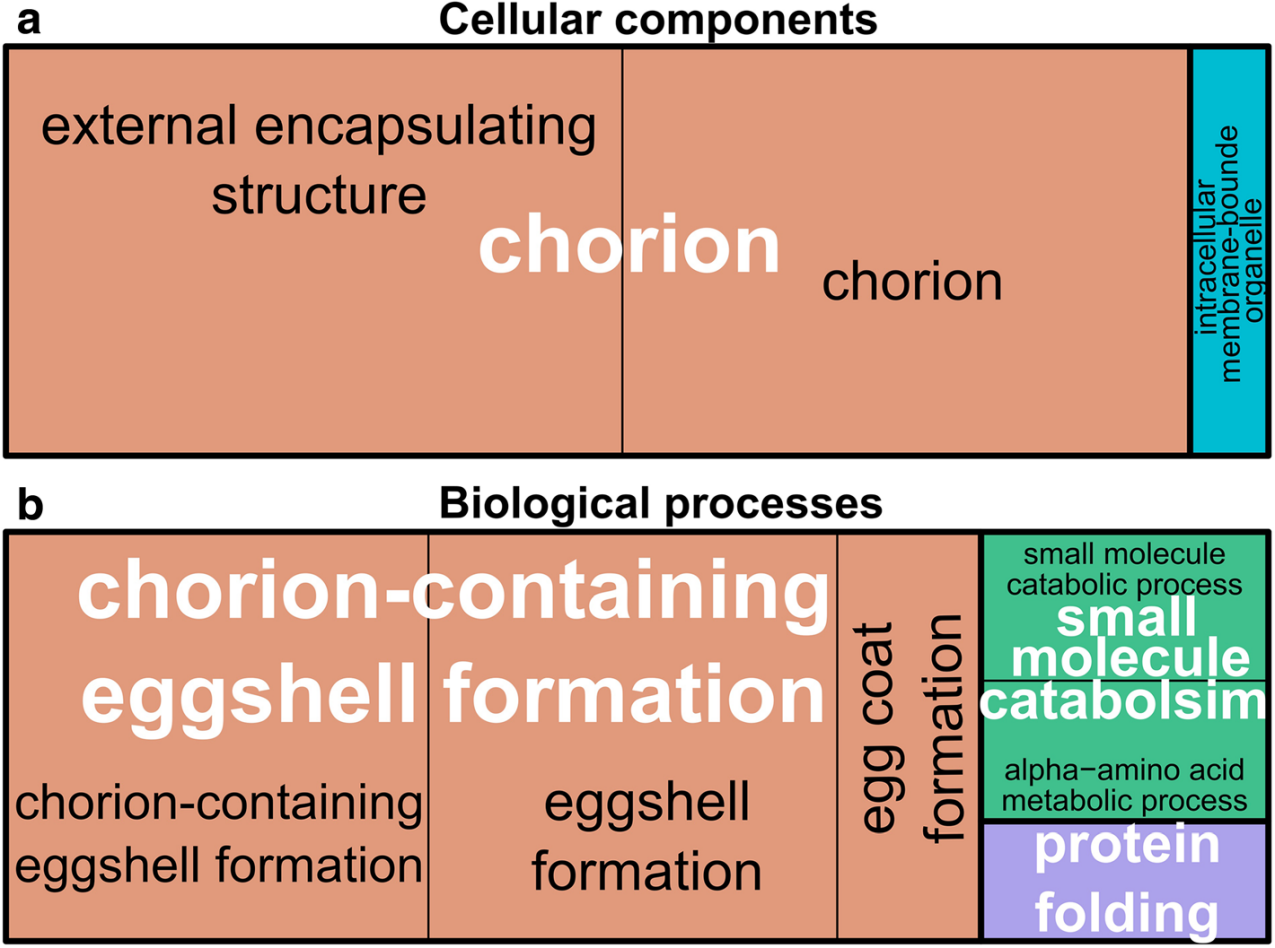
Treemap representation from REVIGO of overrepresented GO-terms from downregulated genes in acclimated flies for (**a**) cellular components and (**b**) biological processes. In each treemap, each rectangle represents a significant GO-term. The sizes of rectangles are adjusted to reflect the relative corrected *p-value* (i.e. the larger the rectangle, the more significantly the GO-term was). Within the treemaps, GO-terms sharing the same color belong to the same GO superclusters whose names are labelled in white

Two strategies are often adopted for enrichment analysis of pathways: the analysis of all differentially expressed genes together or the analysis of up- and downregulated genes separately. The analysis through the separate strategy is supposed to be more reasonable and powerful than the strategy with all differential genes together [41]. Hence, in the present work, GO terms enrichment analyses were performed separately on up- and downregulated gene sets in acclimated flies using GO-TermFinder [42]. For upregulated genes, enrichment analyses resulted in 20 significant GO-terms for cell component. These indicated that regulated genes were mainly located in ‘plasma membrane’ or ‘synapse’. Analyses also detected 26 enriched GO-terms for molecular functions, many of which were redundant and designated enrichment of ‘ion transport’ and ‘signaling across membranes’. Finally, for biological process 30 different, but sometime redundant, GO-terms were enriched. The most significant involved ‘ion transmembrane transport’, ‘response to stimulus’, ‘cell communication’, ‘signal transduction’ and various nervous system processes. ‘Carbohydrate homeostasis’ was also found to be enriched (Fig. 3, Additional file 2: Tables S3 to S5). For the set of downregulated genes, analyses resulted in three significant GO-terms for cell component (i.e., ‘external encapsulating structure’, ‘chorion’, and ‘intracellular membrane-bounded organelle’), no GO-term was enriched for molecular function, and 11 GO-terms were enriched for biological process, including ‘eggshell formation’, ‘vitelline membrane formation’, ‘carboxylic acid catabolic process’ or ‘protein folding’ (Fig. 4, Additional file 2: Tables S6 to S8). Results of these analyses were very similar to outputs obtained with STRING annotation tool [43], which detected similar enriched GO-terms. This latter analysis also found a single enriched KEGG pathway: ‘starch and sucrose metabolism’ (Additional file 2:Tables S9 and S10).

To facilitate interpretations, functional redundancy among GO-terms was reduced, and the presence of superclusters of overrepresented GO-terms was visualized in treemaps using REVIGO program [44]. In the treemaps, representative GO clusters are shown as rectangles whose size reflects the *p*-values. Related GO-terms are then joined into superclusters that present a particular relevance. For cellular component, REVIGO found the following superclusters: ‘integral component of plasma membrane’ and ‘chorion’, for up- and downregulated genes respectively (Figs. 3a and 4a). For molecular functions, one main GO supercluster was found for upregulated genes: ‘ion transmembrane transporter activity’ (Fig. 3b). For biological processes, three superclusters were found from upregulated genes: ‘G-protein coupled receptor (GPCR) signaling pathway’, ‘cation transport’ and ‘system process’ (Fig. 3c). GO-terms related to downregulated genes formed a single supercluster: ‘chorion-containing eggshell formation’ (Fig. 4b).

**Fig. 5:**
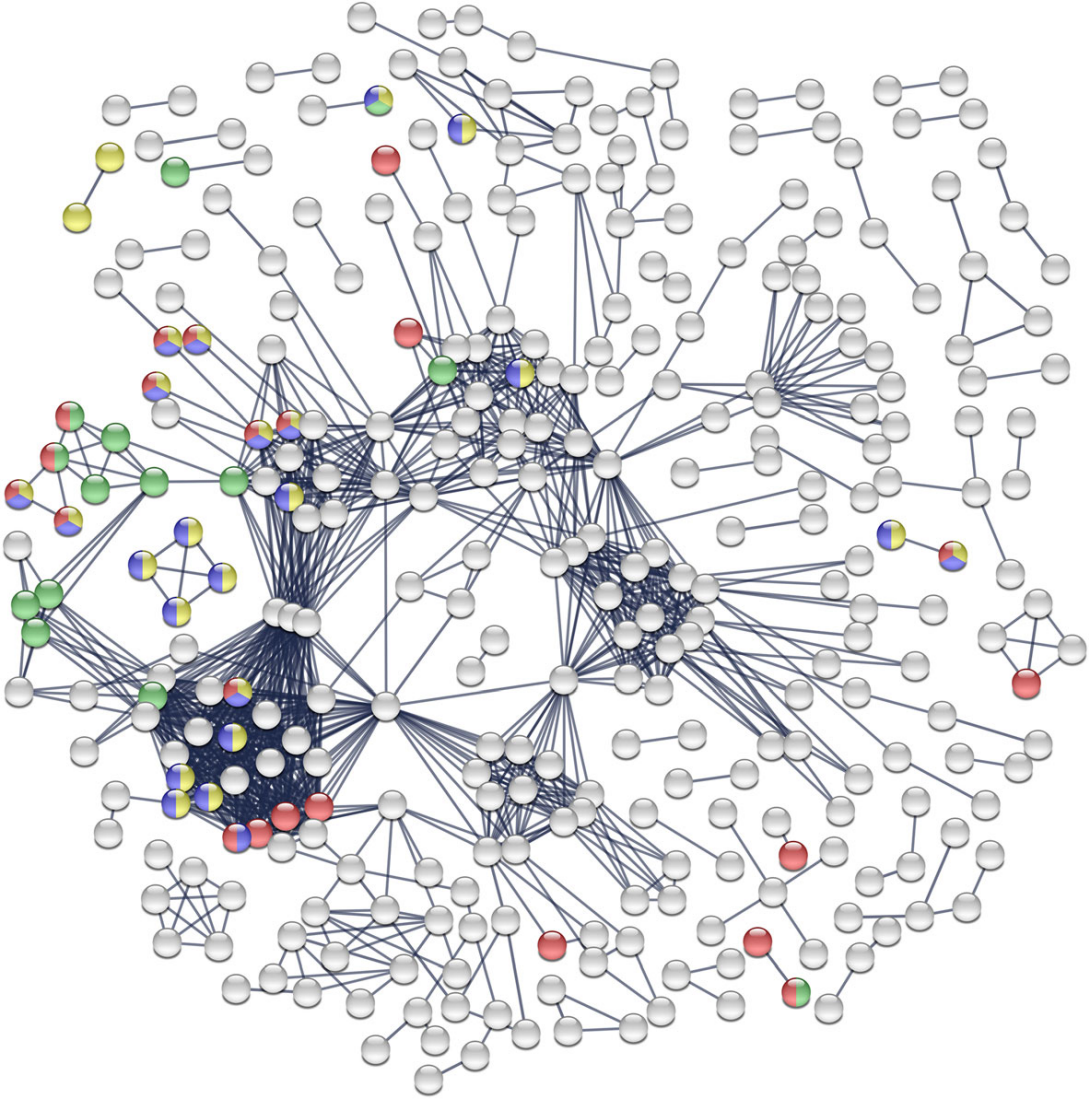
Interaction network resulting from the set of upregulated genes in acclimated flies. Each node represents a protein, and each line represents an interaction between two proteins. The gene sets were analyzed for putative protein-protein interactions using STRING program with default settings, with exception of the confidence interactions value that was set to high confidence (score > 0.9). Disconnected nodes are not represented. Genes involved in the major GO superclusters detected in REVIGO (see Fig. 3) are highlighted with different colors: Cellular component: red: ‘integral component of plasma membrane’; Molecular functions: blue: ‘ion transmembrane transporter activity’; Biological processes: yellow: ‘cation transport’; green: ‘GPCR signaling pathway’

**Fig. 6:**
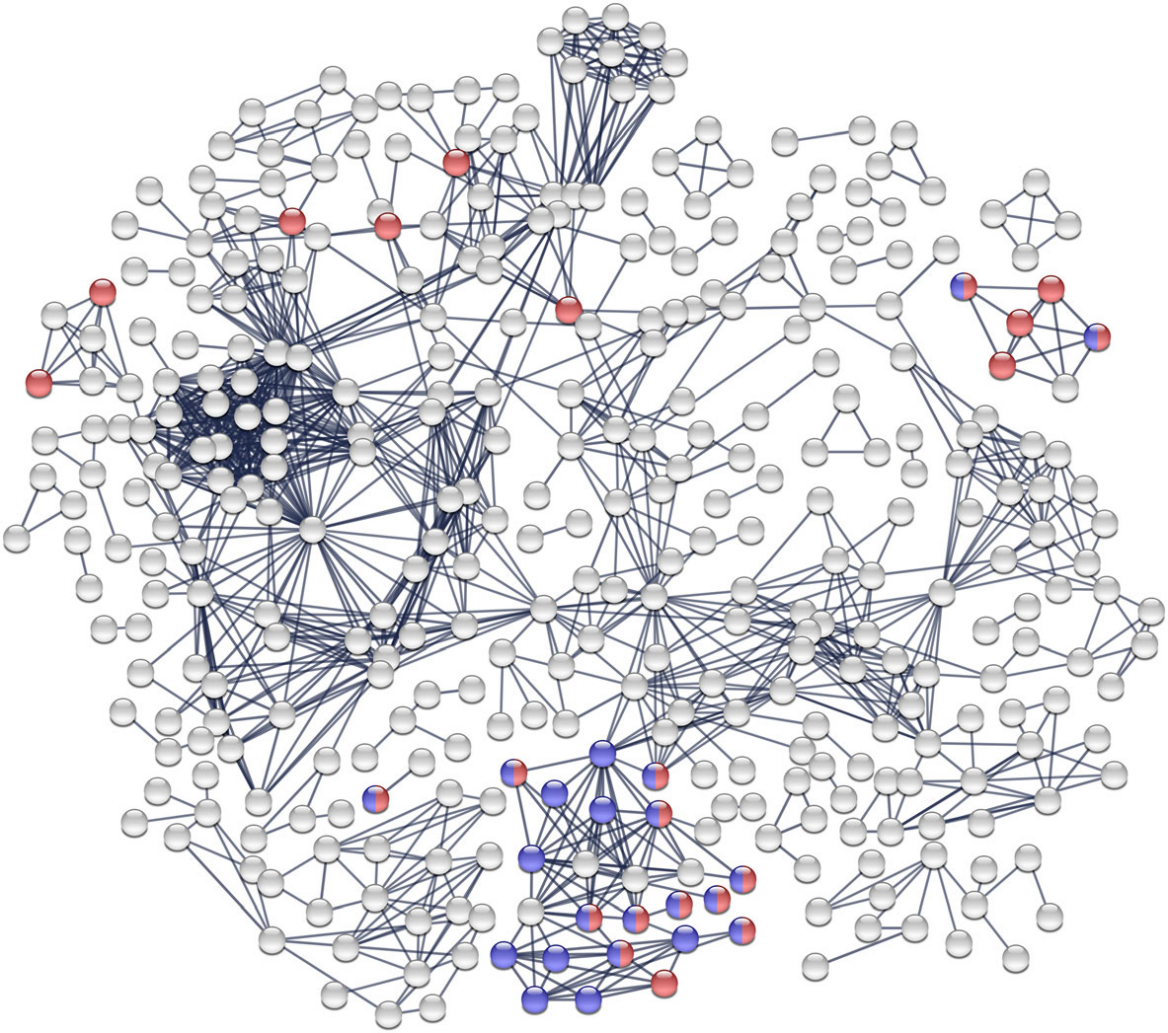
Interaction network resulting from the set of down regulated genes in acclimated flies. Each node represents a protein, and each line represents an interaction between two proteins. The gene sets were analyzed for putative protein-protein interactions using STRING program with default settings, but with high confidence interactions value (score > 0.9). Disconnected nodes are not represented here. The thickness of the edges are related to confidence in data (thicker lines indicate stronger evidence for interaction). Genes involved in the major GO superclusters detected in REVIGO (see Fig. 4) are highlighted in color: Cellular component: blue: ‘chorion’; Biological processes: red: ‘chorion-containing eggshell formation’

Gene Ontology (GO) enrichment analyses were also performed in STRING (considering false discovery rate, i.e. FDR, < 0.05) and provided exactly same output as GO:term finder analyses (these results are available in Additional file 2: Tables S9 and S10). As shown in Figs. 5 and 6, the up and down regulated genes had significant associations and intricate interactions (protein-protein interactions enrichment *p*-value = 1.0e-16 for both up and down regulated genes). This indicates that, in response to acclimation, many functionally related genes were concurrently regulated. Genes involved in the major GO-terms superclusters detected in REVIGO (see Figs. 3 and 4) are highlighted within the networks with different colors (see Figs. 5 and 6 captions) and this revealed that these GO superclusters involved many highly connected genes.

In the present work, we considered genes as differentially expressed when *q.values* (FDR-adjusted *p.value*) were < 0.05. However, classically, differentially expressed genes can be also defined using a fold change (FC) cutoff as a supplementary parameter [45]; however the functional/biological meaning of cutoffs are questionable. To confirm our functional analysis, we performed another GO-terms enrichment analysis on differentially expressed gene sets with a FC cutoff > 1.5. Globally, similar outputs were found, with the same main GO-terms being enriched with or without FC cutoff. These results are available in Additional file 2: Table S12.

## Discussion

### Acclimation increases cold tolerance of *Drosophila suzukii*

We subjected *D. suzukii* adults to incremental acclimation durations to determine cold pre-exposure duration needed to acquire high cold tolerance. Very short acclimation durations (2 or 6 h) at mildly low temperature did not markedly improved cold tolerance (Fig. 1). In previous studies, rapid cold hardening had either no impact [35], or a positive impact [33, 34] on *D. suzukii* cold tolerance. To observe rapid cold hardening response, protocols typically involve pre-exposures to stressful temperatures (around 0 °C) [46]. The temperature used in the present study to acclimate insects (10 °C) was probably not low/stressful enough to trigger a rapid acquisition of cold tolerance. On the other hand, as reported in other insects [47, 48], cold tolerance increased when acclimation duration increased. We found that survival was high and maximum after six days of acclimation and then remained high with longer acclimation durations. Despite acute and chronic survival reached a plateau after six days of acclimation, CCRT and Ct_min_ decreased further after nine days of acclimation. Our results are in accordance with previous findings showing that in *D. suzukii* acclimation at mildly low temperature for several consecutive days deeply promotes adult cold tolerance [25, 35, 38]. We therefore confirm that *D. suzukii* displays high and efficient cold tolerance plasticity; this capability likely contributes to its invasive success in temperate cold regions.

### Activity of ion transmembrane transporters seems important for acclimation

GO-terms enrichment analysis revealed that the major part of upregulated genes were located in cell membranes, neurons and synapses. Molecular functions mainly involved ‘ion transmembrane transporter activity’ and many biological processes involved ‘ionic transporter activity’ (Figs. 3 and 5). Involvement of similar GO-terms has previously been reported in *D. melanogaster* flies that developed at low temperature (13 °C) [49–51]. Multiple genes linked to ‘ion transmembrane transport activities’ were upregulated in cold acclimated flies (Ca^2+^, Na^+^ or K^+^ channels; K^+^ transporters; Na^+^ transporters; ATPase ion transporters; Na^+^/H^+^ transporters; transmembrane organic anion transporters; see Table 3). Genes linked to ion channels are intimately correlated to cold tolerance acquisition in *Gryllus pennsylvanicus* [29]. Under permissive thermal conditions, insects maintain ion homeostasis by compensating the natural leakage of ions across membranes through active transports [13, 19]. Cell membranes are highly thermosensitive macromolecules and temperature decrease induces changes of membrane fluidity, and in extreme cases, transition of membrane phospholipidic bilayer from a liquid crystalline phase to a more rigid lamellar gel phase [20, 52]. These conformation changes can in turn alter ion permeability of membranes [52], provoking loss of ions homeostasis [20, 53]. Functions of membrane-embedded enzymes, proteins and transporters could be altered by these changes in membrane fluidity [52, 54, 55], but also by direct kinetic effects of cold temperatures [19, 31, 56], participating in the deregulation of ion balance [13, 31, 32, 57, 58]. Alteration of ion equilibrium can directly damage cells and tissues [31, 57, 59, 60] and provoke depolarization of membranes, altering action potentials of muscles and neuron cells, conducting to loss of neuromuscular functions and coma [13, 60–67]. In insects, cold tolerance acquisition is correlated with preservation of transmembrane ion balance [31, 57, 64, 68]. This has been reported in *Drosophila* flies [32, 64, 69], including *D. suzukii* [70]. One of the underlying mechanisms of ion balance preservation is likely the plasticity of ion channel thermal sensitivity and the recruitment of ion transporters [13, 19, 64, 69, 71, 72]. The maintenance of ion homeostasis allows electrochemical properties of membranes to be persevered, guaranteeing neuromuscular functions [13, 19, 64]. Our data strongly suggest that cold acclimation in *D. suzukii* induces plastic expression of genes that play essential roles in transport activity of ions across membranes, most likely to prevent disruption of ion homeostasis that may occur at low temperature [70].

**Table 3:** List of genes discussed in the text

Gene	Gene ID^a^	FC^b^	Function or process
*NaCP60E*	DS10_00003598	1.77	Transporters: ion channels
*Ca-alpha1D*	DS10_00000955	1.54	
*KCNQ*	DS10_00002789	1.68	
*Hk*	DS10_00004874	1.51	
*Irk2*	DS10_00011692	2.01	
*Irk3*	DS10_00006400	2.44	
*Vha14–2*	DS10_00012657	Only in CAF	ATPase ion transporters
*Vha68–1*	DS10_00007687	1.44	
*VhaAC39–2*	DS10_00009944	Only in CAF	
*VhaPPA1–2*	DS10_00011222	Only in CAF	
*ppk15*	DS10_00012645	Only in CAF	Sodium transporters
*ppk17*	DS10_00000507	2.49	
*ppk5*	DS10_00010923	Only in CAF	
*ppk9*	DS10_00003499	Only in CAF	
*Oatp30B*	DS10_00001585	1.49	Organic anion transporters
*Oatp33Ea*	DS10_00008440	2.64	
*Oatp33Eb*	DS10_00008442	1.50	
*Oatp58Dc*	DS10_00002075	2.44	
*Nha1*	DS10_00001446	1.38	Sodium:proton transporters
*Nha2*	DS10_00010553	2.23	
*Nhe2*	DS10_00001057	1.90	
*GABA-B-R2*	DS10_00012213	2.08	Neurotransmitter receptors
*nAChRbeta2*	DS10_00012765	2.56	
*nAChRalpha7*	DS10_00004780	1.57	
*DopEcR*	DS10_00010177	2.58	
*5-HT1B*	DS10_00005814	2.44	
*Grip*	DS10_00008207	1.59	
*GluRIIE*	DS10_00012665	2.45	
*mth*	DS10_00007901	Only in CAF	GPCR involved in life span and stress response
*mthl12*	DS10_00003566	2.63	
*mthl14*	DS10_00004634	1.71	
*mthl15*	DS10_00000453	2.07	
*mthl9*	DS10_00004608	1.77	
*Hex-t1*	DS10_00012733	Only in CAF	Carbohydrate metabolism
*Hex-C*	DS10_00005493	1.46	
*Pepck1*	DS10_00005716	1.58	
*AkhR*	DS10_00001466	2.56	Carbohydrate and lipid homeostasis
*Desat1*	DS10_00012265	2.07	Synthesis of unsaturated fatty acids
*Hsc70–2*	DS10_00009454	0.27	Molecular chaperones or co-chaperones
*Hsp60C*	DS10_00006901	0.68	
*Hsp27*	DS10_00003843	0.65	
*HIP-R*	DS10_00006110	0.76	
*Totz*	DS10_00013345	10.86	
*Hsp22*	DS10_00003839	2.66	
*Cp7Fb*	DS10_00006848	0.02	Structural gene for eggshell formation (chorion)
*Cp7Fc*	DS10_00006849	0.04	
*Cp15*	DS10_00003769	0.04	
*Cp16*	DS10_00003771	0.01	
*Cp18*	DS10_00003768	0.00	
*Cp19*	DS10_00003770	0.03	
*Cp36*	DS10_00006850	0.09	
*Cp38*	DS10_00006851	0.09	
*Yp1*	DS10_00004890	0.13	Structural gene for the yolk (Vitellogenin)
*Yp2*	DS10_00004891	0.11	
*Yp3*	DS10_00008400	0.16	
*Vm26Aa*	DS10_00007679	0.11	Oogenesis; vitelline membrane formation
*Vm26Ab*	DS10_00008464	0.20	
*Vm32E*	DS10_00001909	0.11	
*Vm34Ca*	DS10_00001679	0.10	

^a^Spotted Wing Fly Base (http://spottedwingflybase.org/)

^b^Positive and negative values of fold change (FC) for upregulated and downregulated in acclimated flies respectively. *CAF* cold acclimated females, *COF* Control Females

### Genes related to neuronal activity are positively correlated with cold acclimation

Loss of neuromuscular function at low temperature may be linked to impairment of synaptic actions [19]. Indeed neurotransmitter release depends on the activity of Ca^2+^ channels, which may be impaired by depolarization [72] and altered fluidity [73] of membranes at low temperature [52]. Here we observed regulation of genes located in neurons and synapses (Fig. 3a). In addition, several genes coding for neurotransmitter receptors were regulated after cold acclimation (GABA; acetylcholine; dopamine; serotonin; glutamate; Table 3). In cold acclimated *G. pennsylvanicus* several genes linked to neurotransmitters were also upregulated [29], and in diapausing females of *Drosophila montana* maintained for several months at 4 °C, microarrays revealed up regulation of genes involved in dopamine and serotonin synthesis [74]. Furthermore, a previous study that explored transcriptional adjustments in *D. suzukii* showed that the GO-term ‘Neurotransmitter transporter activity’ was also associated with the winter-phenotype [37]. These altered gene expressions are likely related to adjustments of neurotransmitter activities at the synaptic level in order to compensate cold deleterious effects.

### Cold acclimation alters expression of genes involved in cellular signaling

One of the main GO-term superclusters from upregulated genes was ‘G-protein coupled receptor (GPCR) pathway’ that comprised many GO-terms related to signal transduction or signaling (Figs. 3c and 5). ‘Response to stimulus’ and ‘cell communication’ were also highlighted among the most enriched GO-terms. This suggests a major role of genes related to cellular signaling pathways for cold tolerance acquisition. In *D. ananassae*, populations selected for cold tolerance showed upregulation of GO-terms implied in ‘cell communication’ and ‘signaling’ [75]. Interestingly, cold acclimated *G. pennsylvanicus* also showed upregulation of genes linked to ‘GPCR activity’ [29]. Among genes involved in the enriched GO-term ‘GPCR signaling pathway’, we observed several founding members the methuselah family (*mth*, *mthl12*, *mthl14*, *mthl15*, *mthl9*) that were all upregulated in acclimated insects. These genes are *Drosophila* GPCRs involved in the modulation of life span and stress response including heat, starvation, and oxidative damage [76]. GPCRs are transmembrane receptors, initiators of signal transduction and cellular responses, and are involved in a large panel of physiological functions [77]. Stress signaling and thermal plasticity in insects is regulated by protein kinases [78–80], and protein kinase signaling cascades can be activated by GPCRs [81]. Upregulated GO-terms linked to GPCR activity could therefore be linked to a global response or sensing of low temperatures, initiating transduction signal cascades triggering cold acclimation.

We identified two genes coding for G protein-coupled inwardly-rectifying potassium channels (*Irk2* and *Irk3*) that were regulated in cold acclimated flies (see Table 3). These ion channels are primary effectors of GPCR, and participate in hyperpolarization of cell membranes [82]. As previously discussed, ion channel activities are of major importance to counterbalance disturbance of ionic homeostasis due to cold temperatures. Regulation of transcripts linked to GPCR activity could therefore also be linked to ion homeostasis maintenance, through adjustment of ion channels.

### Possible role of carbohydrate metabolism in response to cold acclimation

We expected to observe regulation of candidate genes involved in some of the canonical cold-acclimation mechanisms, such as membrane modifications or cryoprotectant (sugar) metabolism. The GO-term ‘carbohydrate homeostasis’ was indeed enriched in acclimated flies as well as the KEGG pathway ‘starch and sucrose metabolism’. Genes upregulated in acclimated flies included several enzymes (*Hex-t1*, *Hex-C, Pepck1*) playing key roles in carbohydrate metabolism and sugars interconversions. We also noted the upregulation of an adipokinetic hormone receptor (*AkhR*), a GPCR neuropeptide/hormone receptor involved in carbohydrate and lipid homeostasis [83]. We also found upregulation of *desaturase* (*Desat1*), a gene well known to be involved in the synthesis of unsaturated fatty acids [84]. Desaturases play roles in cold-induced phospholipid restructuring [54] and upregulation of *Desat* genes has been correlated with enhancement of cold hardiness [85, 86]. *Desat1 and Desat2* were also reported to be upregulated in diapausing *D*. *montana* females [87], but downregulation of *Desat* genes was reported in cold-acclimated *Drosophila virilis* group species [88]. Profiling with various ‘Omics’ techniques has provided supporting evidence for changes in carbohydrate metabolism and accumulation of sugars (particularly glucose, sucrose, fructose and trehalose) after both rapid and gradual cold acclimation in *Drosophila* [22, 27, 51, 89–91]. Our observation supports the general view that regulation of carbohydrate and lipid metabolism is an element of cold tolerance acquisition in *D. suzukii* [25, 37].

### Minor changes in stress genes expression in response to cold acclimation

We expected to find regulation of genes involved in stress response. Functional annotation revealed ‘protein folding’ as enriched GO-term associated to downregulated genes in acclimated flies. Heat shock chaperones, such as *Hsc70–2, Hsp60C, Hsp27* or *HIP-R* (a co-chaperone), were downregulated in acclimated flies. *Hsc70–2* and *Hsp60C* are constitutively expressed and not known to be cold-responsive [92]. There is a constant need for chaperone assistance during de novo protein folding and refolding of polypeptide chains [93], and hence, the reduced expression of heat shock chaperones in cold acclimated flies may be linked to reduced de novo protein folding at mild low temperature. We found no clear indication of stress genes being upregulated in acclimated flies, except *Hsp22* (FC = 2.6) or *Totz* (FC = 10); the latter was among the most upregulated genes in cold acclimated flies (Fig. 2b, Table 3). *Totz* belongs to *Turandot* family; these genes are part of a humoral stress reaction unlike the heat shock response which mainly deals with the intracellular accumulation of denatured proteins [94]. *Turandot* genes also respond to other types of stress such as heat, UV or oxidative agents like paraquat [94, 95]*. Turandot* genes have also been shown to respond to low temperature likely because cold activates immune pathways [95, 96].

### Apparent decrease in oogenic activity with cold acclimation

Many genes, relatively less expressed in cold acclimated flies, were involved in ‘chorion and eggshell formation’ (forming a main GO supercluster) (Figs. 4 and 6). Genes coding for chorion proteins, yolk proteins or vitelline membrane proteins were among the most downregulated genes in acclimated flies (see Table 3). In *D. melanogaster,* egg production is highest between 18 and 23 °C and is strongly reduced at temperatures below or above this range [97, 98]. Expression of chorion-related genes follows this pattern as highest expression has been reported at intermediate temperatures [49]. The lowest developmental thermal threshold for ovarian maturation is generally around 10–12 °C in temperate drosophilids [99]. In *D. suzukii*, studies reported a reproductive dormancy mainly due to development at low temperature [33, 34, 37]. This reproductive arrest has also been associated with reduced expression of yolk protein gene (*Yp1*) [34]. Following the release of cold-induced dormancy, all yolk proteins transcripts have been found to be upregulated in *D. melanogaster* [100]. Reduced expression of genes related to oogenesis in acclimated *D. suzukii* females suggests a dormancy syndrome. In our experimental design, all flies developed and remained 5 days at 25 °C after eclosion for maturation before treatment; therefore, all females contained mature eggs before they were cold acclimated. The gene patterns we observe here could translate a reduced oogenic activity at low temperature. Interestingly, Lirakis et al.*,* [101] provided a very detailed study of oogenesis in a range of dormancy-inducing conditions in *D. melanogaster*. They reported that one-week old mature flies (maintained at 25 °C) had active oogenesis and mature eggs. When these one-week old mature flies were transferred to dormancy-inducing conditions (10 °C, 10 L: 14D), mature eggs were still present but oogenesis was stopped. Hence, they demonstrated that a dormancy-like phenotype (i.e. block of oogenesis) can be observed in mature flies when exposed to low temperature. In our setting, mature *D. suzukii* flies were acclimated by transferring them from 25 to 10 °C for 9 days. Just as in *D. melanogaster*, this situation could have stopped oogenic activity, explaining the relatively lower expression of genes involved in chorion and eggshell formation. It is not clear whether arrest of oogenesis is a passive consequence of low temperature with no adaptive value for cold tolerance or whether this mechanism is an active protective strategy. Data from drosophila species support that cold-induced oogenesis arrest (via quiescence or diapause) is actually part of an integrated mechanism of cold adaptation and cold stress tolerance mechanism [99, 101].

## Conclusions

This work provides a characterization of transcriptomic changes in response to cold acclimation in *D. suzukii*. Cold tolerance of *D. suzukii* gradually increased with acclimation duration leading to highly cold-hardy phenotype, adding to the body of evidence that this fly possesses high thermal plasticity. We observed major transcriptional remodeling after cold acclimation, primarily involving ion transport and various signaling pathways across membranes and within neuronal parts, and a decrease of the reproductive (oogenesis) function. We suggest these mechanisms represent the core part of the physiological strategy of cold tolerance acquisition in *D. suzukii.* This study provides a list of new candidate genes related to cold tolerance in this fly. In particular, we have highlighted regulation of many genes of interest playing putative roles in ion transport and homeostasis. These processes determine neuromuscular functions, which are highly affected by low temperature, and therefore, constitute the fundamentals of insect cold tolerance [13]. Despite that acclimation treatments used in laboratory settings are not fully ecologically relevant, it is probable that molecular mechanisms similar to those described in the present study may occur in fields during seasonal acclimatization when temperatures progressively drop in the fall. From an applied point of view, cold acclimation may be useful for ongoing sterile/incompatible insect technique programs against *D. suzukii* [9]. After industrial production of males, insects need to be stored at low temperature [102] and cold acclimation could be used to prime insects and extend their shelf life. Insects may also be thermally conditioned before inundative to mitigate thermal stress in fields.

## Methods

### Flies’ rearing conditions and acclimation procedure

The *D. suzukii* line used in this work is a population collected from infested fruits originated from different locations in Trentino (Italia), and brought to the Vigalzano station of the Edmund Mach foundation (46.042574 N, 11.135245E) in 2011. This line was sent to our laboratory (Rennes, France) in early 2016, and has been reared under lab conditions ever since. Flies were reared in glass bottles (100 mL) and supplied ad libitum with a carrot-based diet (for 1 l: agar: 15 g, sucrose: 50 g, carrot powder: 50 g, brewer yeast: 30 g, cornmeal: 20 g, kalmus: 8 g, Nipagin: 8 mL). Flies were kept in incubators (Model MIR-154-PE; PANASONIC, Healthcare Co., Ltd. Gunma, Japan) at 25 °C, 12 L: 12D. Males were identified visually and were manually separated from females with an aspirator without CO_2_ to avoid stress due to anesthesia [103]. Acclimation was induced as follows: 5-day old flies were held either in the rearing conditions (i.e. non-acclimated treatment) or they were cold-exposed at 10 °C in incubators (Model MIR-154-PE; PANASONIC, Healthcare Co., Ltd. Gunma, Japan) for 0 h, 2 h, 6 h, 12 h, 24 h, 48 h, 72 h, 6 days (144 h) or 9 days (216 h), in order to generate nine treatments of incremental acclimation durations. The acclimation temperature of 10 °C was chosen because this temperature is cold enough to induce an acclimation response in *D. suzukii* without causing mortality in adults [25]. Photoperiod during thermal treatments was standardized at 12 L: 12D.

### Cold tolerance assays

#### Acute cold stress

From each of the nine treatment groups males and females were randomly taken and distributed in five replicates of 10 individuals that were submitted to − 5 °C for 60 min, using glass vials immersed in a glycol solution cooled by a cryostat (Cryostat Lauda ECO RE 630). After exposure, flies were allowed to recover in 40 mL food vials under rearing conditions. Survival was assessed by counting the number of dead and living individuals in each vial 48 h after acute cold stress.

#### Chronic cold stress

From each of the nine treatment groups males and females were randomly taken and distributed in five replicates of 10 individuals that were submitted to 0 °C for 24 h, using 40 mL food vials placed in a cooled-incubator (Model MIR-154-PE; PANASONIC, Healthcare Co., Ltd. Gunma, Japan). After exposure, flies were allowed to recover in 40 mL food vials under rearing conditions. Survival was assessed by counting the number of dead and living individuals in each vial 48 h after chronic cold stress.

#### Critical thermal minimum (Ct_min_)

To estimate the Ct_min_ we used a knockdown column consisting of a vertical jacketed glass column (52 × 4.7 cm) containing several cleats to help flies not falling out the column while still awake. In order to regulate the temperature, the column was linked to a cryostat (same model as for acute stress assays), and temperature was checked into the column using a thermocouple K connected to a Comark Tempscan C8600 scanning thermometer (Comark Instruments, Norwich, Norfolk, UK). The thermocouple was inserted at the center of the column, at mid height. Approximately 60 males or females of each conditions were introduced at the top of the column. Flies were allowed to equilibrate in the device at 18 °C for a few minutes, after which the temperature was decreased to 0 °C at 0.5 °C/min. At each fly passing out and falling out of the column the Ct_min_ (°C) was recorded. The experiment ended when temperature reached 0 °C.

#### Chill coma recovery time (CCRT)

CCRT is defined as the resurgence time of motor activity after a cold knockdown [104]. In order to knockout flies, 40 males and females from each 9 treatments were submitted to 0 °C for 12 h, using a food vial placed in a cooled-incubator (same model as chronic stress assays). Directly after exposure, flies were rapidly transferred to a 25 °C regulated room, and spread on a large plane surface using a fine paint brush. Recovery time was individually recorded when a fly was able to stand up. Experimentation ended after 120 min, and non-recovered flies were counted.

#### Statistical analyses

Except for CCRT, all analyses of cold tolerance assays were performed using R (version 3.4.3; R Core Team, 2016). We modeled survival to − 5 °C and 0 °C separately with generalized linear models (GLM) with logistic link function for proportion outcomes (i.e. number of dead/alive flies per vial). Ct_min_ data were analyzed using a GLM following a Gaussian error family with an identity link function. For these models, response variables were dependent on acclimation duration, sex, and the interaction between these two. We analyzed the effect of each variable through an analysis of deviance (“Anova” function in “car” package, [105]). To facilitate interpretation of GLM effects (from acute and chronic cold stress and Ct_min_ data), effect plot function in the package “effects” [106] was used. The effect plots generated show the conditional coefficients (“marginal effects”) for all variables and interaction terms, and are available in Additional file 1: Figure S1.

CCRT data were analyzed using survival analysis with the software Prism (version 5.01; GraphPad, La Jolla, CA, USA). We compared each recovery curves using pairwise comparisons (Gehan-Breslow-Wilcoxon Test) for males and females separately. Alpha level of significance for survival analyses was adjusted thanks to Bonferroni correction (α = 0.0013).

### RNA extraction and sequencing

Transcriptomic analyses were performed only on females. Three replicates of 15 females from the control group and from the 216 h (9 day) cold acclimation group were snap-frozen in liquid N_2_. Samples were ground to a fine powder using pestles in 1.5 mL Eppendorf tubes immerged in liquid N_2_. RNA extractions were performed using a Nucleospin Kit (Macherey Nagel, Düren, Germany) following manufacturer’s instructions. At the end of the process, total RNA was eluted in 50 μL of RNase-free H_2_O. For each sample we measured RNA concentrations using a Nanodrop 1000 (Thermo Scientific, Waltham, MA), and estimate integrity using an Agilent Bioanalyzer nanochip (Agilent, Palo Alto, CA). Bioanalyser outputs are shown in Fig. S3. Next-generation RNA sequencing was performed by Eurofins Genomics (Ebersberg, Germany). RNA strand-specific libraries were created using Illumina TruSeq Stranded mRNA Library Preparation Kit (Illumina) according to the manufacturer’s instructions. Briefly, polyA-RNA was extracted from total RNA using an oligodT-bead based method. After mRNA fragmentation, first-strand and dUTP-based second strand synthesis was carried out, followed by end-repair, A-tailing, ligation of the indexed Illumina Adapter and digestion of the dUTP-strand. Size selection was done using a bead-based method. After PCR amplification, the resulting fragments were cleaned up, pooled, quantified and used for cluster generation. For sequencing, pooled libraries were loaded on the cBot (Illumina) and cluster generation was performed following manufacturer’s instructions. Paired-end sequencing using 125 bp read length was performed on a HiSeq2500 machine (HiSeq Control Software 2.2.58) using HiSeq Flow Cell v4 and TruSeq SBS Kit v4. For processing of raw data RTA version 1.18.64 and CASAVA 1.8.4 were used to generate FASTQ-files. RNAseq produced six libraries: three for control and three for cold acclimated females.

### Mapping, differential expression analyses, gene ontology term enrichment and protein-protein interactions

All bioinformatics analyses were performed using Galaxy (https://galaxyproject.org/use/galaxy-genouest/). First, raw data were trimmed using Trimmomatic (version 0.36 [107]), and their quality was checked using FastQC (version 0.11.2 [108]). Reads were then mapped to *D. suzukii* reference genome [109] using Bowtie2 based Tophat (versions: 2.2.8 and 2.1.1, respectively [110]). The mapping resulted in a mean of 71.11% mapped reads. Reads were then annotated using reference annotation of *D. suzukii* [109] and assembled thanks to Cufflinks (version 2.2.1 [111]), and differential expression of transcripts was computed using Cuffdiff (Cuffdiff is part of Cufflinks, version 2.2.1 [111]). We also used two different pipelines for detecting differential expression, edgeR and Deseq2 [112, 113], in order to validate differential expression of transcripts. Very similar outputs of differiential gene expression were found among the three tested pipelines (data not showed). All following analyses were performed on data from Cuffdiff. Transcript expression was considered significantly different between control and cold acclimated flies when the *q.value* (FDR-adjusted *p.value*) was < 0.05. Up and downregulated genes resulting from Cuffdiff were extracted, converted to *D. melanogaster* orthologs, analyzed for GO-terms enrichment using Go-TermFinder [42], and results were reduced and visualized using REVIGO [44]. To ensure validity and robustness of the results, we performed a second analysis in parallel, using the functional enrichment tool in STRING [43]. We used the lists of differential genes (up and down regulated separately) to query STRING database to search for possible protein-protein interactions [43]. The STRING algorithm links proteins (or genes) into networks based on published functional or informatics-predicted interactions [43]. We used the default parameters, with exception regarding the minimum required interaction score, that we increased to 0.9 (high confidence interactions). Disconnected nodes were not displayed in order to increase visibility of the networks.

### qPCR

To validate RNAseq data, we performed qPCR on selected genes. List of primers are available in Additional file 2: Table S11. For each sample, 500 ng of RNA were reverse transcribed to cDNA using Superscript III first-strand synthesis system (Invitrogen Pty, Thornton, Australia) following manufacturer’s instructions. We targeted 11 genes, down- or upregulated, involved in different processes and functions (see Additional file 2: Table S11 for details), including 2 housekeeping genes (*RP49* and *GAPDH* [114, 115]). *RP49* showed the most stable expression among the different samples, and was then preferred over *GAPDH* as reference gene. A Roche LightCycler® 480 (Roche, Basel, Switzerland) using SybrGreen I mix (Roche) was used to perform qPCRs, following the protocol described in [92]. Relative gene expressions were calculated using the ΔΔCt technique [116]. Expression level of genes resulting from qPCR were then correlated to expression levels resulting from RNAseq using Spearman non-parametric tests and a linear regression in Prism (version 5.01; GraphPad, La Jolla, CA, USA).

## Additional files

Additional file 1: **Figure S1.** Effect plots from GLMs: impact of acclimation duration on acute or chronic cold stress survival and Ct_min_. The plots show the conditional coefficients (“marginal effects”) of all variables included in models as well as effect resulting from the interaction term. The variables are acclimation duration, sex, and their interactions. **Figure S2.** Chill coma recovery time of control flies at two different age (5 and 7 days) and flies acclimated for 9 days. Flies have been submitted to 0 °C for 12 h, and then their individual time to recover from coma was recorded at 25 °C. Each point corresponds to the recovery time of one fly. Full lines: females, dotted lines: males. **Figure S3.** Bioanalyser report on RNA extract from Control (COF) and cold acclimated (CAF) samples (females of *D. suzukii*). Cold acclimation consisted of 5 days old females exposed to 10 °C during 9 days. (Agilent Bioanalyzer nanochip, Agilent, Palo Alto, CA). (PDF 1085 kb)

Additional file 2: **Table S1.** Differential gene expression from Cuffdiff: Upregulated genes in acclimated flies. **Table S2.** Differential gene expression from Cuffdiff: Down-regulated genes in acclimated flies. **Table S3.** Gene ontology term enrichment, Up regulated Cell component. **Table S4.** Gene ontology term enrichment, Up regulated Molecular function. **Table S5.** Gene ontology term enrichment, Up regulated Biological process. **Table S6.** Gene ontology term enrichment, Down regulated Cell component. **Table S7.** Gene ontology term enrichment, Down regulated Molecular function. **Table S8.** Gene ontology term enrichment, Down regulated Biological process. **Table S9.** Outcomes from STRING enrichment analysis on upregulated genes. **Table S10.** Outcomes from STRING annotation on down regulated genes. **Table S11.** List of primers used in qPCR. (XLSX 1022 kb)

## Abbreviations

CAF: Cold acclimated females
CCRT: Chill coma recovery time
COF: Control females
Ct_min_: Critical thermal minimum
DD: Degree days
FC: Fold change
FDR: False discovery rate
GLM: Generalized linear models
GO: Gene ontology
GPCR: G-protein coupled receptor
RNAseq: RNA sequencing
