# Supplementary material for: Cold acclimation triggers major transcriptional changes in Drosophila suzukii
Source: BMC Genomics. 2019 May 22;20:413. doi: 10.1186/s12864-019-5745-7 (PMC6532241; doi:10.1186/s12864-019-5745-7)

Cold acclimation triggers major transcriptional changes in *Drosophila suzukii*

Thomas Enriquez[1] ; Hervé Colinet[1]

[1]Univ Rennes, CNRS, ECOBIO - UMR 6553, 263 Avenue du Général Leclerc, 35042 Rennes, France

***Corresponding author:**

Hervé Colinet, Université de Rennes, CNRS, ECOBIO - UMR 6553, 263 Avenue du Général Leclerc, 35042 Rennes, France. Tel: +33 (0)2 23 23 64 38; email: herve.colinet@univ-rennes1.fr

Supplementary Figure S1: page 1
Supplementary Figure S2: page 2
Supplementary Figure S3: page 3

Supplementary Figure S1: Effect plots from GLMs: impact of acclimation duration on acute or chronic cold stress survival and Ct_{min}. The plots are showing the conditional coefficients ("marginal effects") of all variables included in models as well as effects resulting from the interaction terms. The variables are Acclimation duration, Sex, and their interactions.

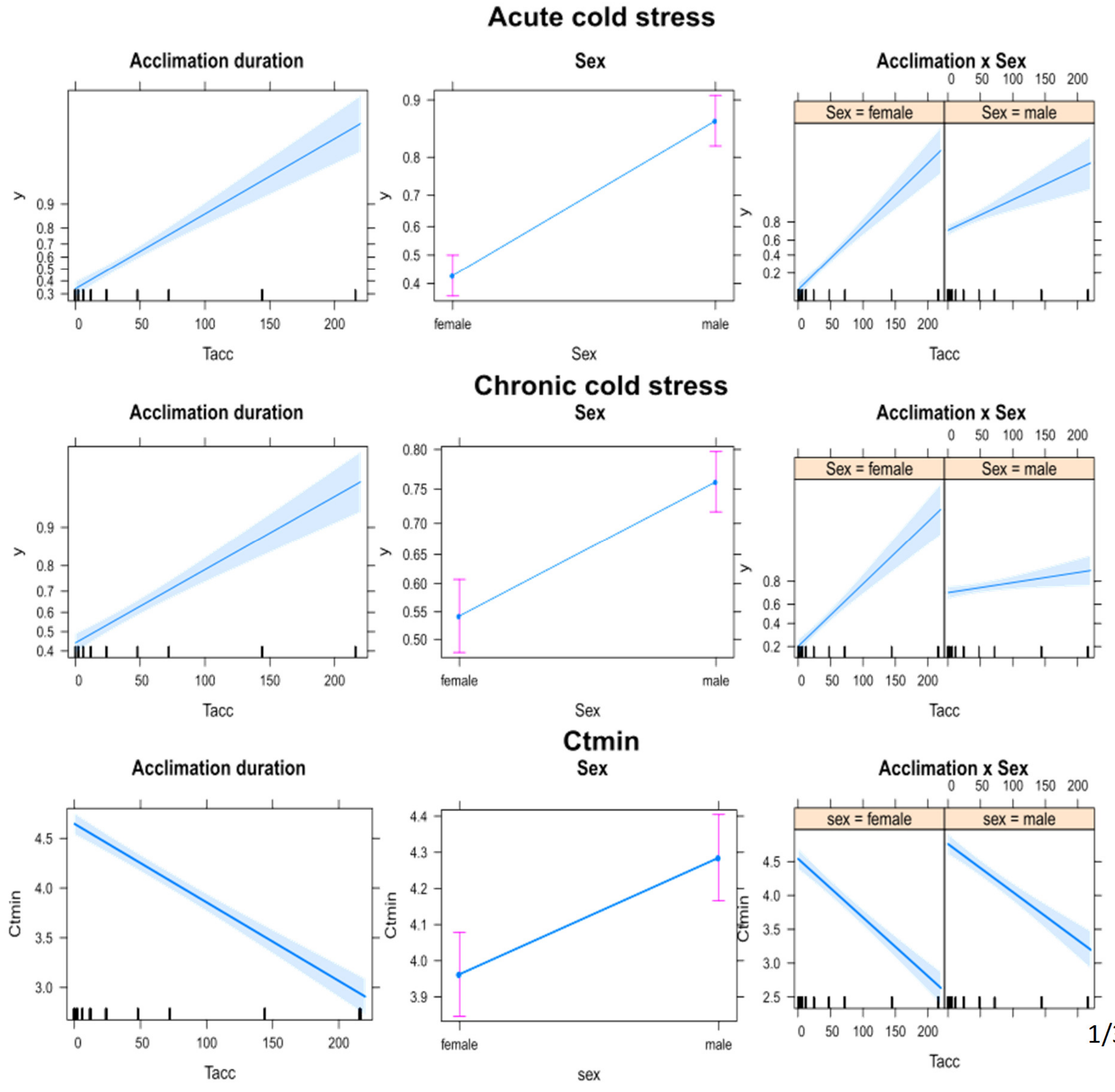

Supplementary Figure S2: Chill coma recovery time of control flies at two different age (5 and 7 days) and flies acclimated for 9 days. Flies have been submitted to 0°C for 12 h, and then their individual time to recover from coma was recorded at 25°C. Each point corresponds to the recovery time of one fly. Full lines: females, dotted lines: males.

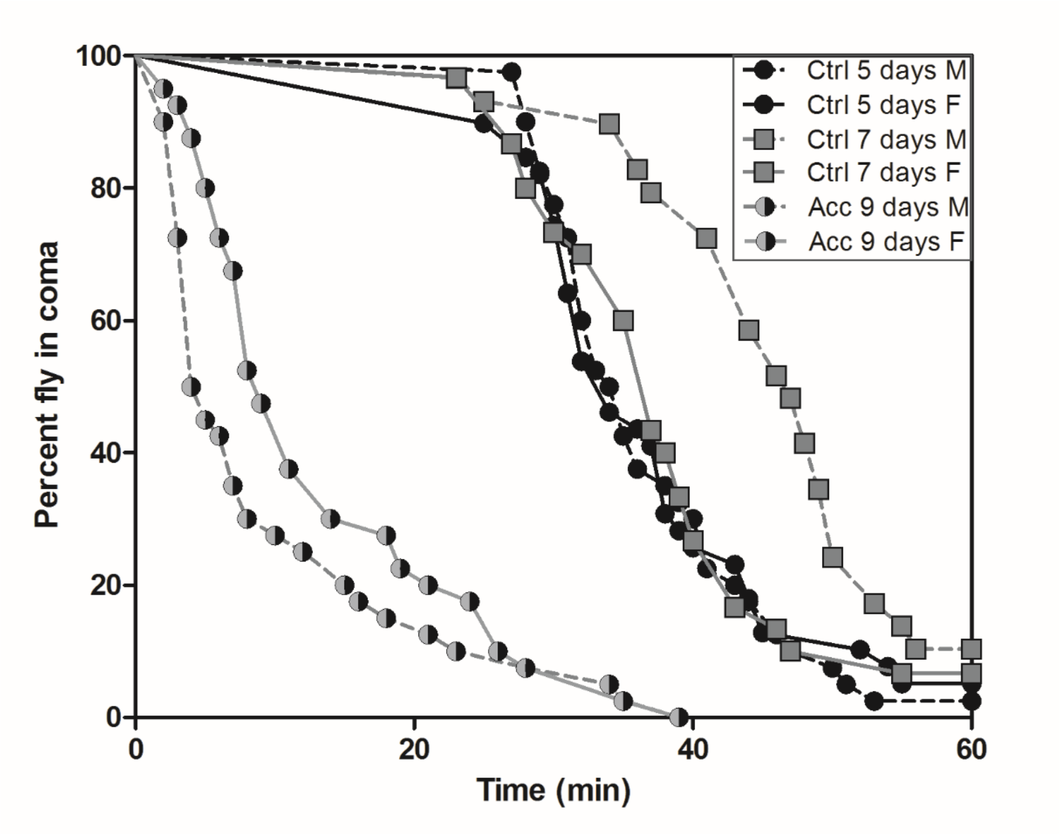

Supplementary Figure S3: Bioanalyser report on RNA extract from Control (COF) and cold acclimated (CAF) samples (females of *D. suzukii*). Cold acclimation consisted of 5 days old females exposed to 10°C during 9 days. (Agilent Bioanalyzer nanochip, Agilent, Palo Alto, CA).

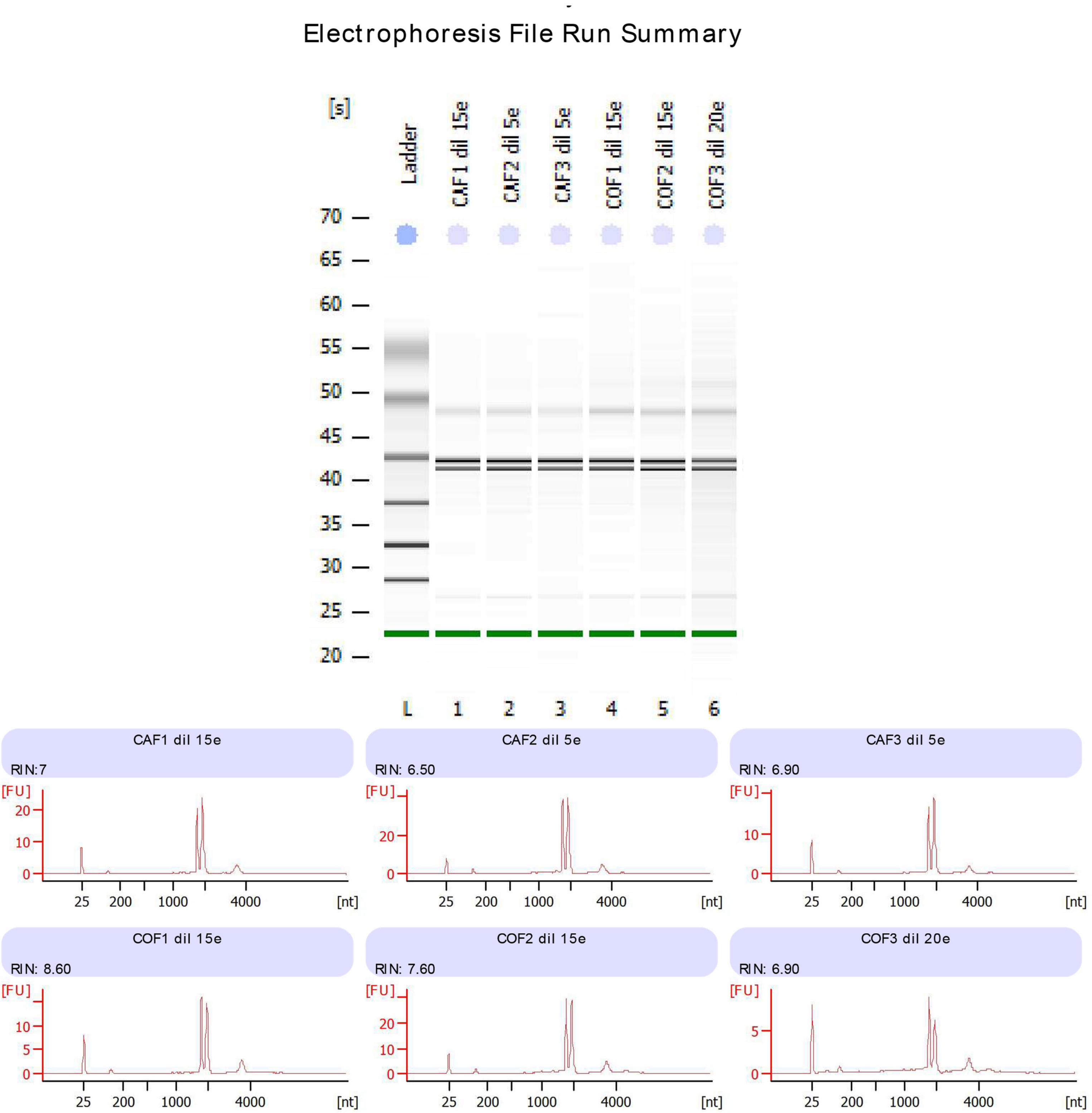

Supplement: Supplementary file 1 — Figure S1. Effect plots from GLMs: impact of acclimation duration on acute or chronic cold stress survival and Ctmin. The plots show the conditional coefficients (“marginal effects”) of all variables included in models as well as effect resulting from the interaction term. The variables are acclimation duration, sex, and their interactions. Figure S2. Chill coma recovery time of control flies at two different age (5 and 7 days) and flies acclimated for 9 days. Flies have been submitted to 0 °C for 12 h, and then their individual time to recover from coma was recorded at 25 °C. Each point corresponds to the recovery time of one fly. Full lines: females, dotted lines: males. Figure S3. Bioanalyser report on RNA extract from Control (COF) and cold acclimated (CAF) samples (females of D. suzukii). Cold acclimation consisted of 5 days old females exposed to 10 °C during 9 days. (Agilent Bioanalyzer nanochip, Agilent, Palo Alto, CA). (PDF 1085 kb) [file 12864_2019_5745_MOESM1_ESM.pdf]
